# Case Report: Renal artery stenosis in children: ultrasound as a decisive diagnostic and therapy-accompanying technique!

**DOI:** 10.3389/fped.2023.1251757

**Published:** 2023-11-23

**Authors:** Maire Brasseler, Ilja Finkelberg, Carsten Müntjes, Metin Cetiner

**Affiliations:** ^1^ Department of Paediatrics I, Neonatology, Paediatric Intensive Care, Paediatric Infectiology, Paediatric Neurology, University of Duisburg-Essen, Essen, Germany; ^2^ Department of Paediatrics II, Paediatric Nephrology, Gastroenterology, Hepatology, Transplantation, Endocrinology and Sonography, University of Duisburg-Essen, Essen, Germany; ^3^ Department of Paediatrics III, Paediatric Oncology, Pneumology, Cardiology and Rheumatology, University of Duisburg-Essen, Essen, Germany

**Keywords:** pediatrics, renal artery stenosis, ultrasound, diagnostic, case report

## Abstract

**Introduction:**

Renal artery stenosis in children is rare, and the recommended diagnostic algorithm, including techniques such as catheter-based angiography, CT angiography, magnetic resonance angiography, and ultrasound, is controversial in pediatric cohorts.

**Case presentation:**

We report a case of an 11-year-old girl with renal artery stenosis in whom ultrasonography played a decisive role in confirming the diagnosis and accompanying therapeutic percutaneous transluminal renal artery angioplasty.

**Conclusion:**

Improved ultrasound techniques and the examiner’s experience contribute to improving renal artery stenosis diagnosis in children. In particular, localized sensitive blood flow velocity analysis indicates the advantages of ultrasound compared to other imaging modalities in renal artery stenosis. Therefore, ultrasound should be a focus of future study designs addressing the search for the best diagnostic algorithm.

**Summary:**

The advantages of ultrasound techniques in pediatric patients with renal artery stenosis compared to other imaging modalities are highlighted.

## Introduction

The etiology of arterial hypertension in children is more frequently secondary to underlying renal or endocrinological causes, in contrast to adults with a higher proportion of essential hypertension. Renovascular lesions, mainly caused by fibromuscular dysplasia, comprise approximately 10% of pediatric hypertension cases and are more commonly located in the second and third renal artery branches compared to adults with a predominant location in the main renal artery (1). The preferred diagnostic algorithm [including catheter-based angiography, CT angiography (CTA), magnetic resonance angiography (MRA), and ultrasound], especially in pediatric cohorts, is still controversial. Ultrasound is a non-invasive, radiation-free diagnostic method. The following diagnostic criteria suggest a renovascular lesion: (1) directly determined stenosis, (2) parvus et tardus waveform, and (3) pathological age-dependent flow parameters (2). Herein, we report a pediatric case of renal artery stenosis using the ultrasound technique in decisive diagnostic, intervention-accompanying, and post-intervention evaluating roles.

## Case description (diagnostic assessment, therapeutic intervention, and outcome)

**Figure 1:**
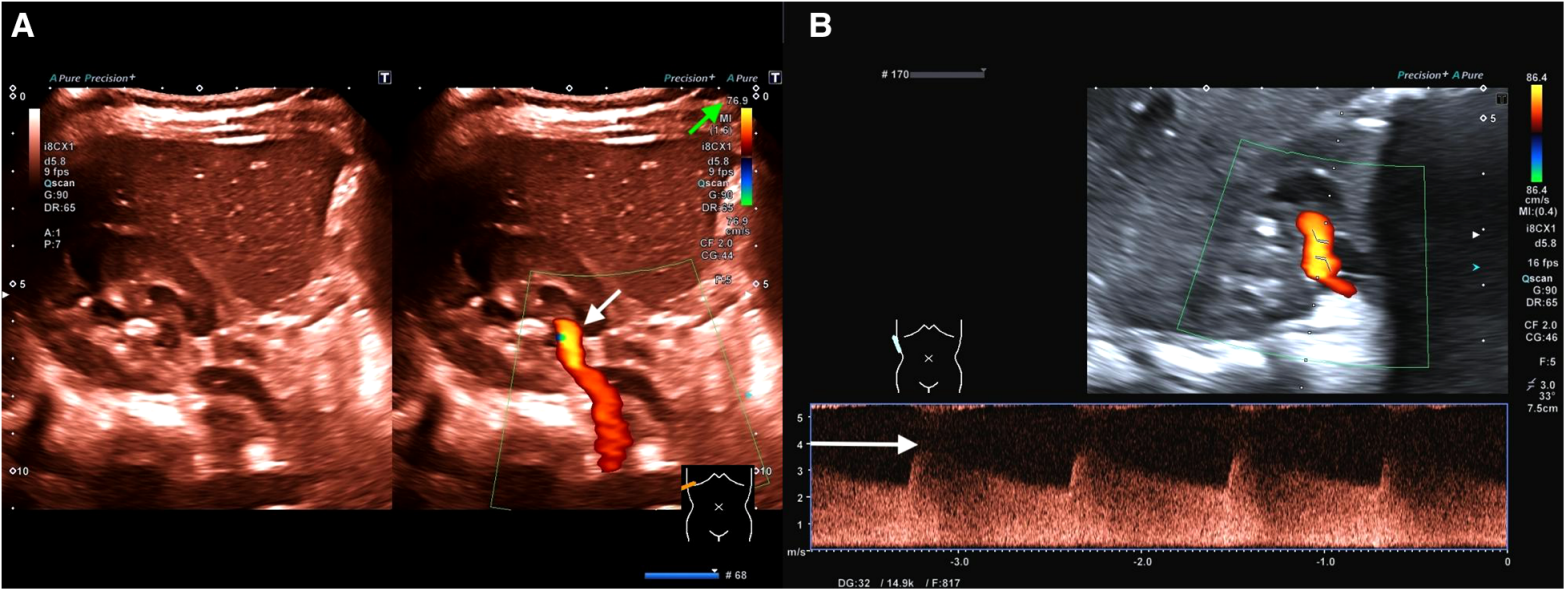
(**A**) (Representation of the kidney in the transversal plane) Color change (brighter orange color; see white arrow) due to significant acceleration of flow velocity in the first renal artery branch caused by stenosis (including Supplementary Video 1). To focus on areas with accelerated flow velocities, flow velocity scaling (see the green arrow) is adjusted to velocities above 76.9 cm/s as a screening method. Only perfusion velocities above this level are illustrated. (**B**) (Representation of the kidney in the longitudinal plane) Flow analysis of the detected stenosis demonstrates a maximum flow velocity of 400 cm/s (see the white arrow; diastolic flow 250 cm/s) accompanied by a decreased resistance index (RI) of 0.38 (normal range of RI: 0.6–0.8).

**Figure 2:**
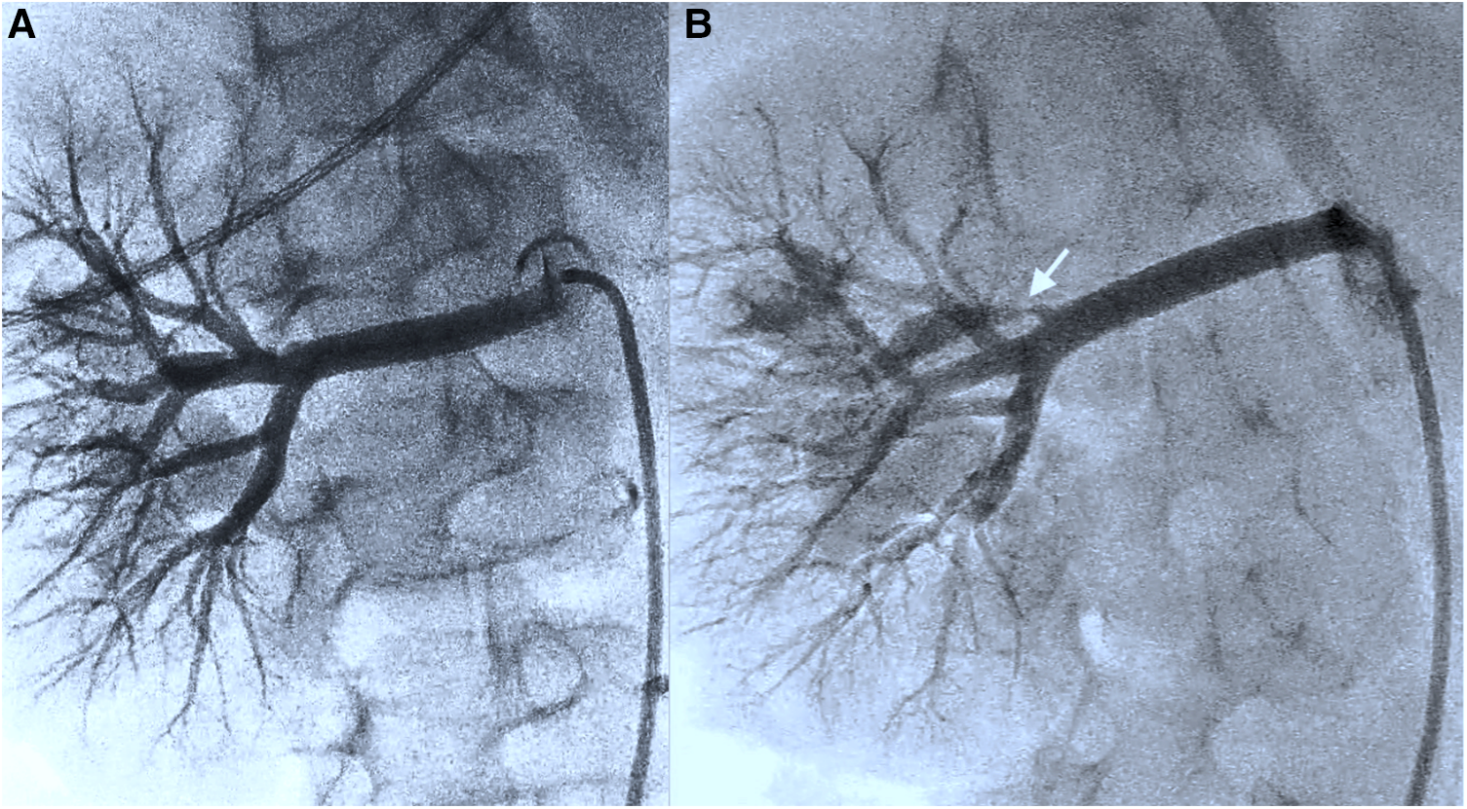
(**A**) (Representation of the kidney in the transversal plane) Initial angiography presentation and further investigation following a change of viewpoints do not detect any stenoses in the renal vascular architecture (including Supplementary Video 2). (**B**) After renewed ultrasound reassurance of stenosis, an additional viewpoint (cranial angulation 30°) reveals thin stenosis in the first renal artery branch (see the white arrow) (including Supplementary Video 3).

**Figure 3:**
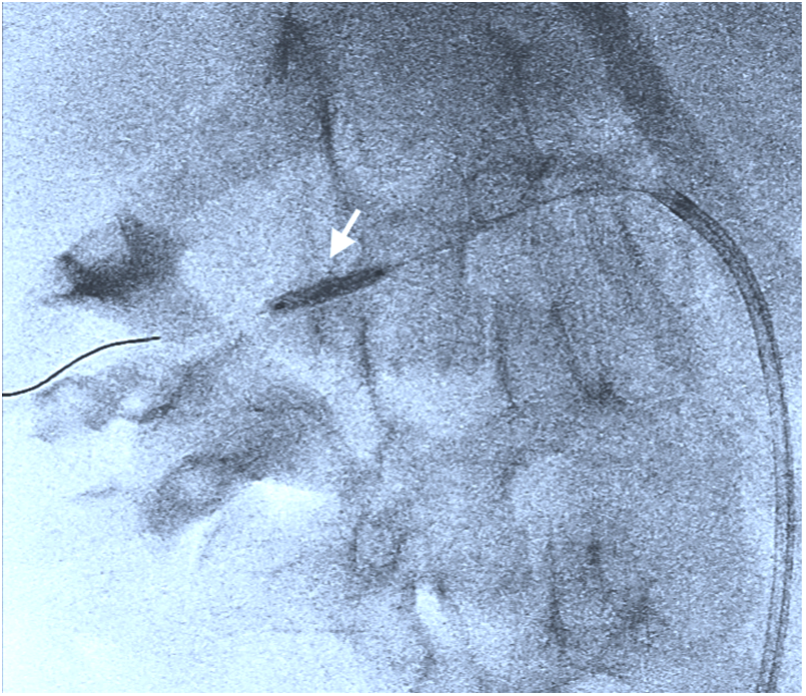
Therapeutic balloon angioplasty of the stenotic area with dilatation of up to 2.5 mm (including Supplementary Video 4).

**Figure 4:**
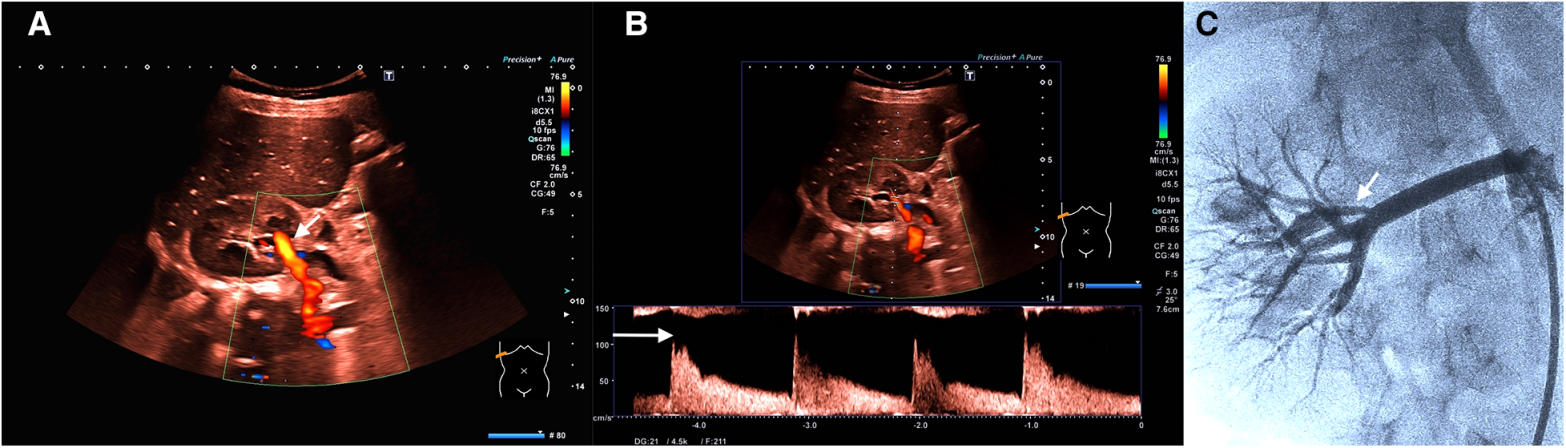
(**A**) (Representation of the kidney in the transversal plane) Color change (less bright; see white arrow) due to deceleration of flow velocity (after intervention with dilatation of stenosis). The same scaling settings as in Figure 1A. (**B**) (Representation of the kidney in the transversal plane) Flow analysis after intervention demonstrates a sharp decrease of maximum flow velocity to 120 cm/s (see the white arrow; diastolic flow 40 cm/s) and a normalization of the RI (0.67). (**C**) Angiography presentation (see the white arrow; the same setting as in Figure 2B) confirms successful therapy (including Supplementary Video 5).

An 11-year-old girl with arterial hypertension presented to our pediatric nephrological outpatient clinic. The girl reported headaches twice a week for 4 months. Clinical examination of the girl showed the absence of pathological murmur in the kidney region, and blood pressure was elevated during day and night (mean 139/94 mmHg, 24 h measurement, 15 mmHg > 99th percentile). Echocardiography excluded aortic stenosis and cardiac hypertrophy, and laboratory examinations showed normal values in creatinine, metanephrine, and endocrinological results; in particular, renin (31.7 ng/L) and aldosterone (163.3 ng/L) were in the normal range. An ultrasound examination showed normal renal parenchyma with a regular corticomedullary differentiation and normal size with a total kidney volume of 158 ml (right 78 ml, left 80 ml). Doppler ultrasound revealed stenosis of a small segmental artery of the right kidney, verified by an arterial flow velocity acceleration of up to 4 m/s (Figures 1, 2, Supplementary Videos 1–3). A therapy with amlodipine (2.5 mg twice daily) and metoprolol (23.75 mg twice daily) was started, and catheter-based angiography with an option for renal angioplasty was initiated. Standard angiography setting angles (anterior and posterior projection and following caudal angulation 30°) could not detect the stenosis. After renewed ultrasound demonstration, an additional setting viewing (cranial angulation 30°) indicated a narrow stenosis (minimal diameter <1 mm) hiding behind the main renal artery in previous viewpoints. Percutaneous transluminal renal artery angioplasty using balloon dilatation (dilatation up to 2.5 mm) was performed without any complications (Figure 3, Supplementary Video 4). Post-intervention, same-day results showed a widened diameter of 2 mm and a declined flow velocity to 120 cm/s in the former segmental artery stenosis (Figure 4, Supplementary Video 5). An immediate decrease of average blood pressure values of about 20 mmHg and a reduction of renin (2.1 ng/L) and aldosterone (<37 ng/L) levels below the normal range after 12 h could be observed. Antihypertensive therapy was terminated; antithrombotic prophylaxis with heparin was administered for 24 h, and aspirin prophylaxis continued for 6 months. Right kidney volume increased to 87 ml (2 weeks after intervention) and 100 ml (11 months later). Follow-up after 4 and 11 months demonstrated age-appropriate blood pressure values (mean 112/69 mmHg, 24 h measurement) in our case (see the timeline in the Supplementary Material).

## Discussion

Current debates on superior performance of CTA vs. catheter-based angiography regarding diagnostic sensitivity and specificity of pediatric renal artery stenosis are ongoing. MRA is evaluated with limited performance due to a difficulty in the detection of intrarenal arterial branches and the necessity of sedation in younger children (3). At present, angiography is considered the gold standard for identifying and treating renal artery stenosis (4). Trautmann et al. reported an undetected stenosis in 5 of 127 patients by computer tomography. In these cases, stenosis occurred in the main renal artery, with additional stenosis in one of the branch arteries. Ten artery stenoses could not be detected by magnetic resonance tomography. All these diagnostic tools focus on direct visualization. Ultrasound technology is frequently evaluated with inferior grading due to the suggested shortcomings in analyzing all sections of the main renal artery and detecting and examining segmental and deeper renal arteries or small accessory renal arteries in their entirety. Our case highlights the strengths of ultrasound and the shortcomings of the aforementioned techniques, including angiography. Only the ultrasound technique can measure flow velocities and indicate thin, small segmental artery stenosis in a visually convincing manner due to the accelerated unusual high flow velocity. In contrast, the anatomical visualization of the stenosis by angiography did not succeed initially and needed ultrasound assurance due to the small caliber of the stenotic artery compared to the unaffected main renal artery. In addition, successful intervention reduced previous normal renin and angiotensin levels below the normal range, emphasizing the need to set normal values in relation to the clinical situation. Ultrasound technology is improving regarding image resolution and perfusion evaluation, including microvascular imaging that can map the smallest vessels with flow velocities beyond 1 cm/s. In addition to excluding renal parenchyma abnormalities, ultrasound combines direct visualization and velocity measurements (5). The examiner’s education remains the most challenging obstacle; involving experienced pediatric ultrasound specialists who assist in examinations using ultrasound live-streaming, as performed preliminarily in our center on a trial basis, might be a promising approach.

## Conclusion

Improved ultrasound techniques and examiner’s experience contribute to improving renal artery stenosis diagnosis in children. In particular, localized sensitive blood flow velocity analysis indicates its advantages compared to other imaging modalities in the case of renal artery stenosis. Therefore, ultrasound should be focused on in future study designs addressing the search for the best diagnostic algorithm.

### Patient perspective

Avoiding lifelong intake of antihypertensive medication was feasible after intervention. Secondary damages due to resolved primary cause of arterial hypertension were prevented. Follow-up evaluation due to a recurrence rate of 20% is essential (6, 7).

## Data Availability

The original contributions presented in the study are included in the article/**Supplementary Material**. Further inquiries can be directed to the corresponding authors.

## Supplementary material

SUPPLEMENTARY VIDEO 1

Color change (brighter orange color; see the white arrow) due to significant acceleration of flow velocity in the first renal artery branch caused by stenosis. To focus on areas with accelerated flow velocities, flow velocity scaling (see green arrow) is adjusted to velocities above 76.9 cm/s as a screening method. Only perfusion above this level is illustrated.

SUPPLEMENTARY VIDEO 2

Initial angiography presentation and further investigation following a change of viewpoints do not detect any stenoses in the renal vascular architecture.

SUPPLEMENTARY VIDEO 3

Finally, after renewed ultrasound reassurance of stenosis, an additional viewpoint from the bottom-up reveals thin stenosis in the first renal artery branch (see the white arrow).

SUPPLEMENTARY VIDEO 4

Therapeutic balloon angioplasty of the stenotic area with dilatation of up to 2.5 mm.

SUPPLEMENTARY VIDEO 5

Angiography presentation (see the white arrow; the same setting as in Figure 2B) confirms successful therapy.

SUPPLEMENTARY DATA SHEET 1

Timeline of the case report.
