# Supplementary material for: Case Report: Renal artery stenosis in children: ultrasound as a decisive diagnostic and therapy-accompanying technique!
Source: Front Pediatr. 2023 Nov 23;11:1251757. doi: 10.3389/fped.2023.1251757 (PMC10701517; doi:10.3389/fped.2023.1251757)
Supplement: Supplementary file 6 [file Datasheet1.pdf]

Supplementary material

Timeline of the Case report

10/2021

Headache twice a week

02/2022

Presentation in the pediatric nephrology outpatient clinic at the University of Duisburg Essen

Ultrasound examination:

stenosis of small segmental artery of the right kidney

arterial flow velocity accelaration up to 4 m/s

Laboratory diagnostics:

normal values for creatinine, metanephrine and endocrinological results, in particular renin (31.7 ng/l) and aldosterone (163.3 ng/l) in the normal range

Therapy:

amlodipine (2x 2.5 mg daily) and

metoprolol (2x 23.75 mg daily)

06.09.2023

Percutaneous transluminal renal artery angioplasty (balloon dilatation up to 2.5 mm)

Post-intervention same day ultrasound: Flow velocity decline to 120 cm/s in the former segmental artery stenosis

07.09.2023

Normalization of blood pressure values and termination of antihypertensive therapy

Reduction of renin (2.1 ng/l) and aldosterone (<37 ng/l) levels below the normal range

Antithrombotic prophylaxis with heparin administered for 24 hours + Aspirin prophylaxis for the following 6 months

01+08/2023

Follow Up

Normal blood pressure

Normal flow velocity speed in former segmental artery stenosis
